# Molecular Epidemiology of *Mycobacterium tuberculosis* Complex in Singapore, 2006-2012

**DOI:** 10.1371/journal.pone.0084487

**Published:** 2013-12-18

**Authors:** Leo Kang-Yang Lim, Li Hwei Sng, Wah Win, Cynthia Bin-Eng Chee, Li Yang Hsu, Estelle Mak, Arul Earnest, Marcus Eng-Hock Ong, Jeffery Cutter, Yee Tang Wang

**Affiliations:** 1 Singapore Tuberculosis Elimination Programme, Tan Tock Seng Hospital, Singapore, Singapore; 2 Department of Pathology, Singapore General Hospital, Singapore, Singapore; 3 Centre for Infectious Diseases Epidemiology and Research, Saw Swee Hock School of Public Health, Singapore, Singapore; 4 Centre for Quantitative Medicine, Graduate Medical School, Singapore, Singapore; 5 Department of Emergency Medicine, Singapore General Hospital, Singapore, Singapore; 6 Ministry of Health, Singapore, Singapore; Institut Pasteur de Lille, France

## Abstract

**Background:**

Tuberculosis remains common in Singapore, increasing in incidence since 2008. We attempted to determine the molecular epidemiology of *Mycobacterium tuberculosis* complex (MTC) isolates locally, identifying major circulating genotypes and obtaining a glimpse of transmission dynamics.

**Methodology:**

Non-duplicate MTC isolates archived between 2006 and 2012 at the larger clinical tuberculosis laboratory in Singapore were sampled for spoligotyping and MIRU-VNTR typing, with case data obtained from the Singapore Tuberculosis Elimination Program registry database. Isolates between 2008 and 2012 were selected because of either multidrug-resistance or potential epidemiological linkage, whereas earlier isolates were randomly selected. Separate analyses were performed for the early (2006-2007) and later (2008-2012) study phases in view of potential selection bias.

**Principal Findings:**

A total of 1,612 MTC isolates were typed, constituting 13.1% of all culture-positive tuberculosis cases during this period. Multidrug-resistance was present in 91 (5.6%) isolates – higher than the national prevalence in view of selection bias. The majority of isolates belonged to the Beijing (45.8%) and EAI (22.8%) lineages. There were 347 (30.7%) and 133 (27.5%) cases clustered by combined spoligotyping and MIRU-VNTR typing from the earlier and later phases respectively. Patients within these clusters tended to be of Chinese ethnicity, Singapore resident, and have isolates belonging to the Beijing lineage. A review of prior contact investigation results for all patients with clustered isolates failed to reveal epidemiological links for the majority, suggesting either unknown transmission networks or inadequate specificity of the molecular typing methods in a country with a moderate incidence of tuberculosis.

**Conclusion:**

Our work demonstrates that Singapore has a large and heterogeneous distribution of MTC strains, and with possible cross-transmission over the past few years based on our molecular typing results. A universal MTC typing program coupled with enhanced contact investigations may be useful in further understanding the transmission dynamics of tuberculosis locally.

## Introduction

Tuberculosis remains an infectious disease of global public health importance, causing significant morbidity and mortality even in developed countries. The capability to genotype *Mycobacterium tuberculosis* complex (MTC) isolates has resulted in greater capacity in delineating previously unknown transmission networks as well as identifying populations at higher risk for the transmission of tuberculosis [1,2]. In the right context, genotyping may also be used to improve contact investigations and evaluate the success of tuberculosis programs [3]. Genotyping using spoligotyping [4] and 15- to 24-loci MIRU-VNTR [5] have been shown to be a useful proxy for demonstrating transmission of tuberculosis [6,7], although they have considerably lower discriminatory power compared to whole genome sequencing and may not define the actual transmission chains of each MTC clone [8].

Singapore is a modern city-state with a first-world healthcare system and escalating migrant and tourist populations [9]. There was a dramatic increase of 26% in the country’s population between 2000 and 2010, occurring primarily in the migrant work force [9]. This was mirrored by the incidence of tuberculosis starting to increase again from 2008, after a sustained fall from a height of 57.1 per 100,000 population in 1998 to a historic low of 35.0 per 100,000 population in 2007 [10]. In 2011, there were 1,533 new cases of tuberculosis among Singapore residents, with an incidence rate of 40.5 cases per 100,000 population [11] – a 15.7% increase compared to 2007 [10]. The number of non-residents on long-stay passes with newly diagnosed with tuberculosis was 593 in 2011 – also the highest for the past few decades [10]. 

The reasons for this rise in incidence of tuberculosis in Singapore are not well understood. Various hypotheses have been put forward, including greater population mobility and community transmission, patient and healthcare system delays in achieving the diagnosis of tuberculosis, and an increasing number of elderly with multiple co-morbidities that render them more vulnerable to disease reactivation [10-12] or infection. However, there is insufficient knowledge about tuberculosis transmission dynamics in Singapore to adequately address the issue. 

The specific aim of this study was to determine the molecular epidemiology of MTC isolates in Singapore, identifying major circulating lineages and clones and obtaining a glimpse of transmission dynamics over the recent years. A previous study had demonstrated that the majority (54.9%) of local tuberculosis isolates belonged to the Beijing lineage [13], and given a rise in the influx of migrant workers from other parts of Asia where the dominant tuberculosis lineage was not the Beijing lineage [9], we were interested to see if this had changed. In addition, a predominance of isolates clustered by MIRU-VNTR and spoligotyping would suggest – although not be proof conclusive given the lack of discriminatory power of these methods when re-evaluated by whole genome sequencing [8,14] – of significant local transmission, whereas the reverse result would suggest the rise in incidence of tuberculosis is primarily due to reactivation of latent disease and importation.

## Materials and Methods

### Study population and isolates

Archived MTC isolates from the Central Tuberculosis Laboratory (CTL) at the Singapore General Hospital (SGH) were tested. Only the first isolate from each patient diagnosed with clinical tuberculosis was selected for genotyping. CTL is one of two microbiology laboratories with MTC culture facilities in Singapore, and process approximately 75% of all tuberculosis cultures in the country. There were two phases of isolate selection and testing in view of funding limitations. Between 2006 and 2007, genotyping was attempted for all viable and culturable archived MTC isolates. Between 2008 and 2012, MTC isolates were genotyped if they were considered to be linked based on prior epidemiological investigations of patient contacts, or if they were multidrug-resistant (MDR-TB). Limited household contact investigations – in the form of formal invitations for contact screening – are sent to household contacts of all patients with culture-positive pulmonary tuberculosis in Singapore. More extensive contact and epidemiological investigations are routinely performed for individuals with drug-resistant tuberculosis or those housed in institutional or correctional facilities.

Demographic data including age, gender, ethnicity and Singapore resident status (defined as being either a citizen or permanent resident) were obtained from the Singapore Tuberculosis Elimination Programme (STEP) registry database [10]. Tuberculosis is a notifiable disease under Singapore law.

### Identification of MTC and susceptibility testing

MTC isolates were cultured using the Mycobacteria Growth Indicator Tube 960 system (BACTEC MGIT 960, Becton Dickinson Microbiology Systems, U.S.A.) and identified using the AccuProbe (Gen-Probe, U.S.A). Susceptibility testing to first-line anti-tuberculosis drugs was also performed on the BACTEC MGIT 960. Multidrug-resistance was defined as resistance to isoniazid and rifampicin.

### Genotyping

Genomic DNA from all viable isolates was extracted using a previously described heat kill method [15]. Spoligotyping was performed using commercial kits following the manufacturer’s instructions (Ocimum Biosolutions, India), as was 24-loci MIRU-VNTR typing (Genoscreen, France).

### Data analysis

The spoligotyping and MIRU-VNTR typing results were combined and analyzed using the categorical coefficient on Bionumerics 5.0 (Applied Maths NV, Belgium), with similarity trees constructed using the unweighted pair group method with arithmetic averages (UPGMA). Minimum spanning tree analysis based on MIRU-VNTR combined with spoligotyping results were constructed using the categorical coefficient. A cluster is defined as isolates from two or more patients with identical MIRU-VNTR and spoligotyping results. Separate analyses to determine clustering were performed for both the earlier (2006-2007) and later (2008-2012) phases, as well as for both phases combined.

Derivation of tuberculosis lineages was performed through submission of MIRU-VNTR and spoligotyping data to http://www.miru-vntrplus.org and use of the available online tools for comparison analysis [16].

The contact investigations database of the STEP registry was reviewed for all patients with clustered isolates in order to determine the proportion of cases with a prior history of tuberculosis contact as well as to determine the presence of any epidemiological links.

Intercooled Stata 10.2 (StataCorp, U.S.A) was used for all other statistical calculations, with level of significance set at 5%. Because of the differences in isolate selection in both phases of testing, comparative analyses were performed between patients in the earlier phase (2006-2007) and those in the later phase (2008-2012) to assess the degree of selection bias. For each phase, comparative analyses were further performed between patients with isolates that were clustered and those with isolates that were not. Dichotomous variables were analyzed with the χ^2^ test or Fisher’s exact test appropriately, and continuous variables were analyzed with the Mann-Whitney U test.

### Ethics

The study was approved by the National Healthcare Group Domain Specific Review Board E, with waiver of consent requirements in view of the retrospective nature of the study (NHG DSRB Ref: 2012/01991).

## Results

During the earlier (2006-2007) and later (2008-2012) phases, there were a total of 3,987 (3,268 culture-positive) and 13,908 (9,068 culture-positive) non-duplicate tuberculosis cases notified to STEP. It is routine practice for the microbiology laboratories in Singapore to perform drug susceptibility testing on at least one MTC isolate cultured from each patient, and there were 39 and 127 MDR-TB cases reported to STEP – comprising 1.2% and 1.4% of all culture-positive cases – during the earlier and later phases. 

**Figure 1:**
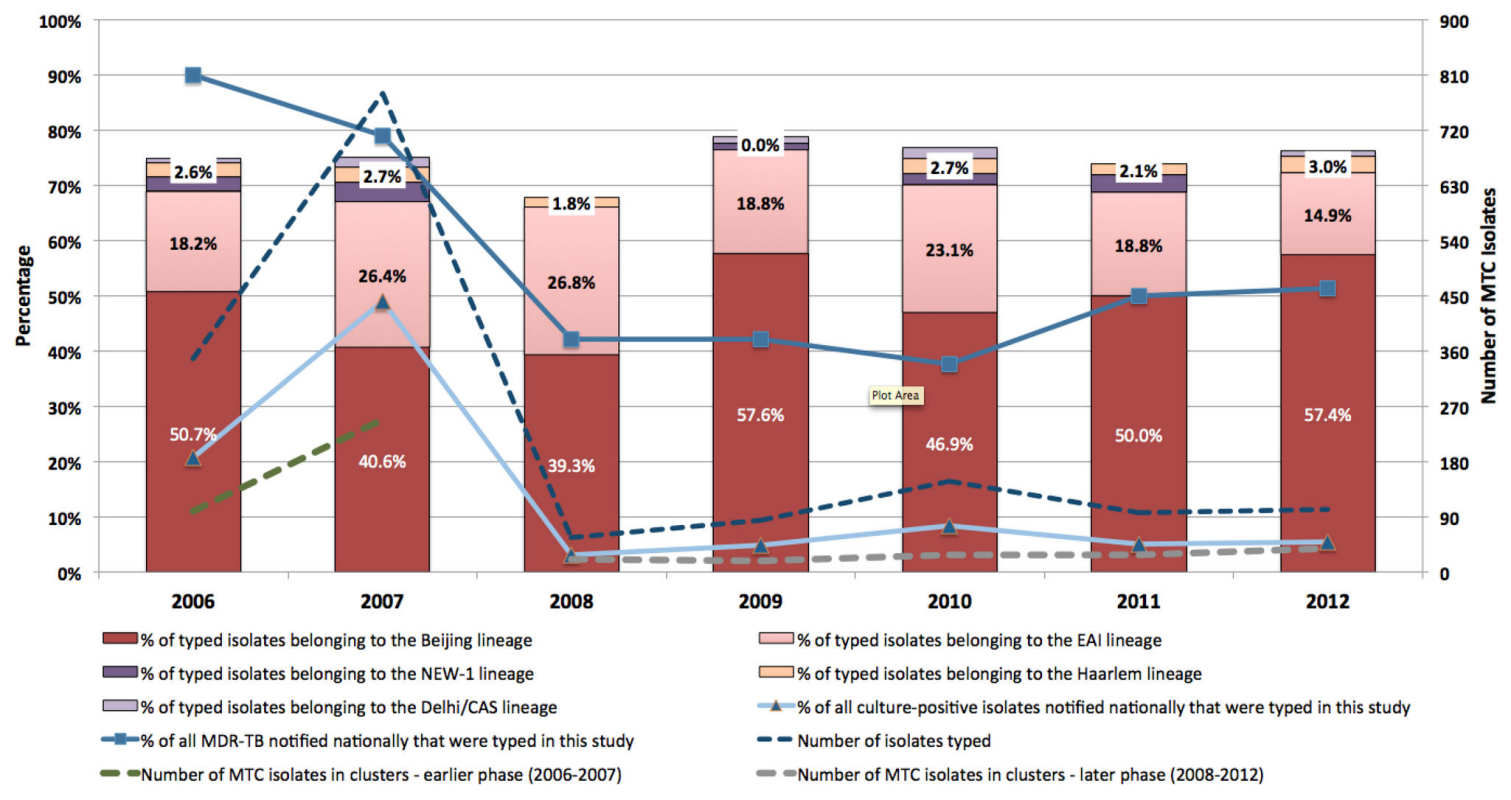
Distribution of MTC isolates typed each year, including the number of isolates clustered via combined spoligotyping and MIRU-VNTR, and the proportion of major tuberculosis lineages. The percentages of all nationally notified culture-positive and multidrug-resistant MTC isolates typed each year are also shown.

There were 1,612 MTC isolates successfully recovered from the CTBL archives with unequivocal results on spoligotyping and MIRU-VNTR typing, with 1,128 isolates from the earlier phase. These comprised 28.3% and 3.5% of all notified tuberculosis cases, and 49.5% and 8.0% of culture-positive cases during the earlier and later phases respectively. The distribution of patient demographics and major lineages by phase of testing is shown in Table 1. There was no significant difference in any of the demographic variables between patients in both phases, although expectedly a higher proportion of isolates in the latter phase were multidrug-resistant. The majority of patients were of Chinese ethnicity, male, and classified as Singapore residents. There were 10 and 9 major lineages for the earlier and later phases respectively, with the majority of isolates belonging to the Beijing and EAI lineages. A significantly higher proportion of isolates in the later phase belonged to the Beijing lineage as opposed to non-Beijing lineages (*p*=0.009). Pearson’s correlation coefficient for the similarity matrices generated by spoligotyping alone and the combination of spoligotyping and MIRU-VNTR for the determination of lineage was 80.1% using the “congruence of experiments” feature in Bionumerics 5.0 (Applied Maths NV, Belgium). The proportion of major tuberculosis lineages and multidrug-resistance by year is shown in Figure 1.

**Table 1:** Distribution of patient demographics, susceptibility testing results and major spoligotypes according to phase of testing (2006-2007 vs. 2008-2012).

**Characteristic**	**Earlier phase 2006-2007** (n = 1,128)	**Later phase 2008-2012** (n = 484)	***p*-value**
Median age, years (IQR)	49 (32-63)	47 (30-63)	0.267
Age distribution:			0.442
0-14 years (%)	4 (0.4)	4 (0.8)	
15-64 years (%)	866 (76.8)	372 (76.9)	
>64 years (%)	258 (22.9)	108 (22.3)	
Male gender (%)	758 (67.2)	344 (71.1)	0.125
Ethnicity:			0.136
Chinese	679 (60.2)	297 (61.4)	
Indian	87 (7.7)	26 (5.4)	
Malay	171 (15.2)	64 (13.2)	
Others	191 (16.9)	97 (20.0)	
Singapore resident (%)	779 (69.1)	321 (66.3)	0.279
Multidrug-resistant isolate (%)	33 (2.9)	58 (12.0)	<0.001
Molecular profiling^1^			
Number of clusters	102	42	N.A.**2**
Patients clustered (%)	347 (30.8)	133 (27.5)	0.186
Lineage:			0.061
Beijing (%)	493 (43.7)	246 (50.8)	
EAI (%)	269 (23.8)	98 (20.2)	
NEW-1 (%)	36 (3.2)	7 (1.4)	
Haarlem (%)	30 (2.7)	10 (2.1)	
CAS (%)	17 (1.5)	5 (1.0)	

^1^ Isolates were clustered based on identical spoligotyping and MIRU-VNTR profiles.

^2^ N.A. = not applicable.

**Figure 2:**
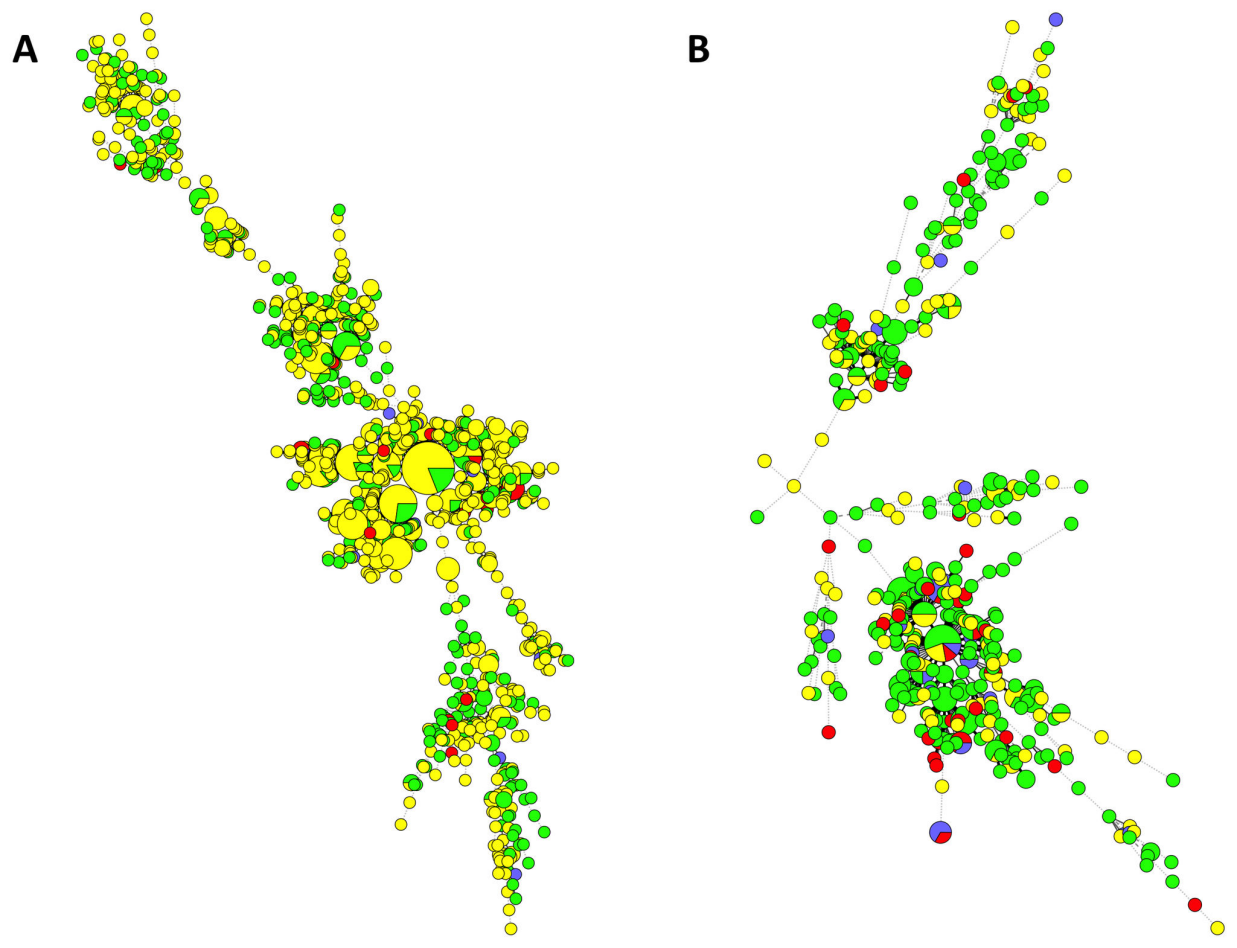
Minimum spanning trees of MTC isolates typed via both spoligotyping and MIRU-VNTR, categorized according to patient resident status and presence of multidrug-resistance (MDR) (yellow = resident with non-MDR-TB, green = non-resident with non-MDR-TB, blue = resident with MDR-TB, red = non-resident with MDR-TB). Each circle represents a genotype and the sizes of the circles are proportionate to the number of members in each group. The distance between circles represents how closely related the different genotypes are to each other. 1A) MTC isolates from the earlier 2006-2007 phase. 1B) MTC isolates from the later 2008-2012 phase.

**Figure 3:**
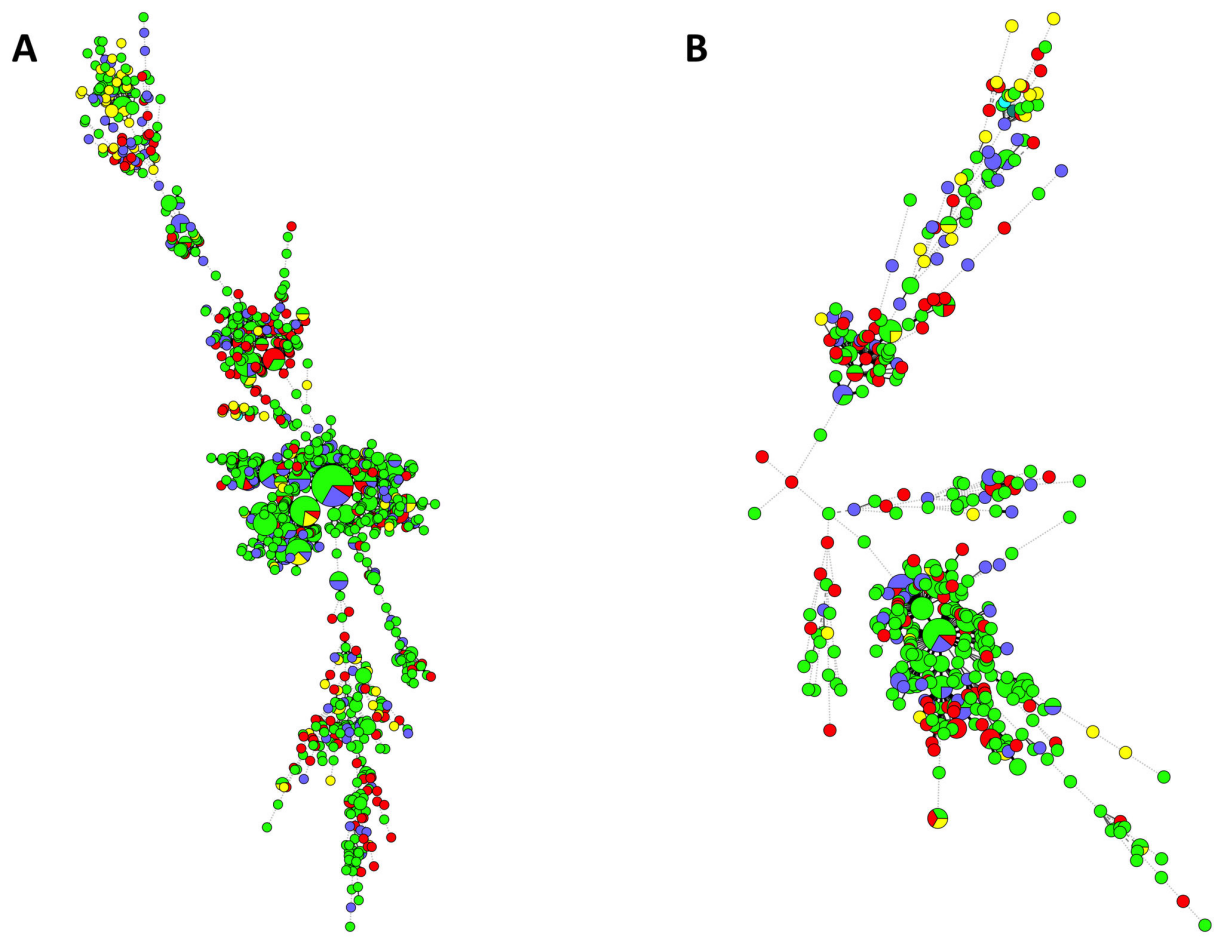
Minimum spanning trees of MTC isolates typed via both spoligotyping and MIRU-VNTR, categorized according to patient ethnicity (yellow = Indian, green = Chinese, blue = Malay, red = others). Each circle represents a genotype and the sizes of the circles are proportionate to the number of members in each group. The distance between circles represents how closely related the different genotypes are to each other. 2A) MTC isolates from the earlier 2006-2007 phase. 2B) MTC isolates from the later 2008-2012 phase.

**Figure 4:**
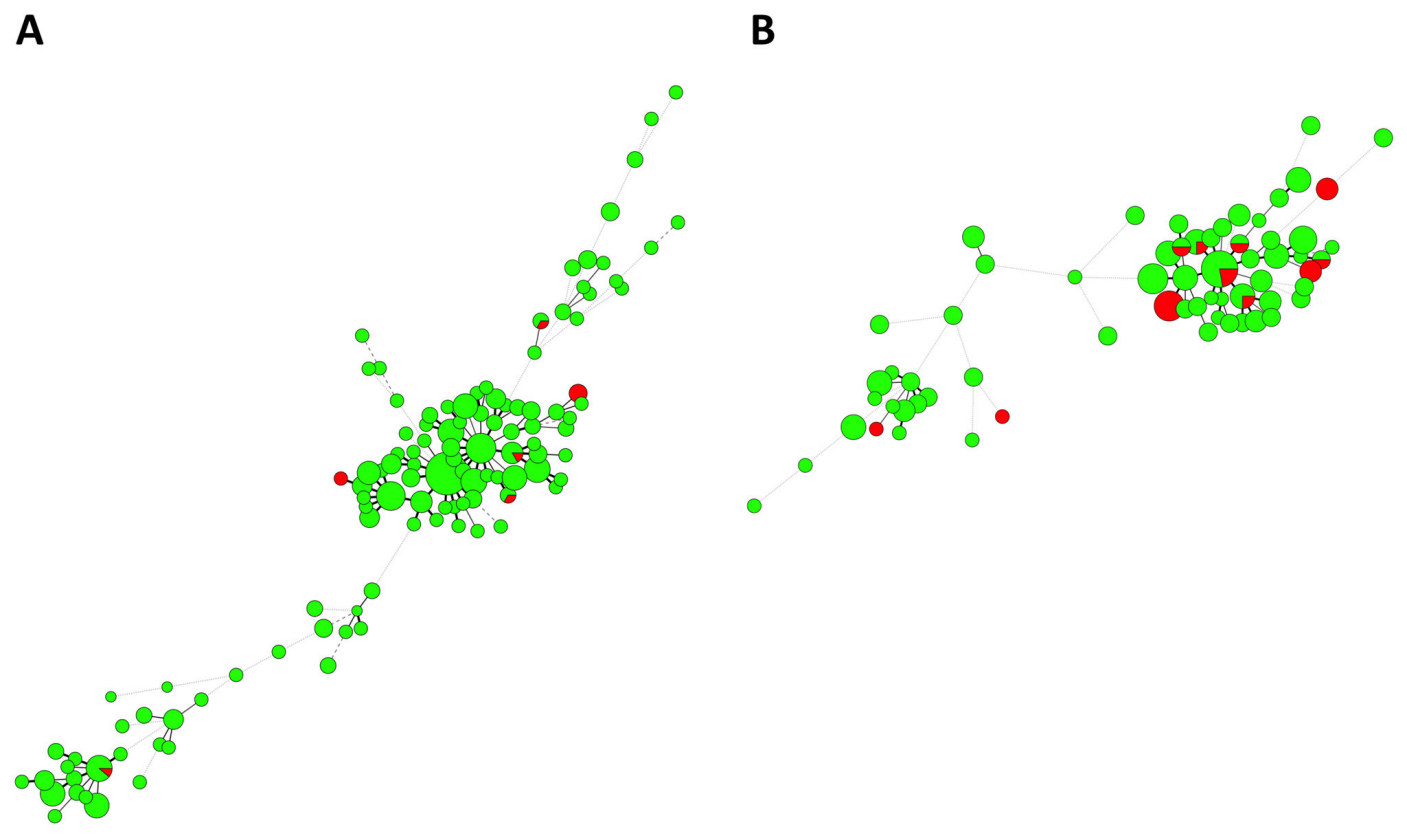
Minimum spanning trees of clustered MTC isolates categorized according to presence of MDR (green = non-MDR MTC, red = MDR MTC). Each circle represents a genotype and the sizes of the circles are proportionate to the number of members in each group. The distance between circles represents how closely related the different genotypes are to each other. 3A) MTC isolates from the earlier 2006-2007 phase. 3B) MTC isolates from the later 2008-2012 phase.

The combined spoligotyping and MIRU-VNTR typing results are displayed in Figures 2 to 4 respectively, stratified according to Singapore resident status, ethnicity and multidrug-resistant MTC. For the earlier phase, there were 347 isolates that were grouped into 102 clusters with identical typing results, whereas 133 isolates were grouped into 42 clusters for the later phase (Table S1). There were 5 clusters with 10 or more isolates/patients in the earlier phase, with the largest cluster comprising 21 isolates. Isolates belonging to the Beijing lineage constituted 4 clusters – the last comprised isolates belonging to the EAI lineage. For the later phase, only 1 cluster had 10 or more isolates (11 isolates), also belonging to the Beijing lineage. Although the majority of patients in smaller (<10 patients) MIRU-VNTR clusters belonged to one ethnicity only, multi-ethnic representation was seen in virtually all the larger clusters. The majority of large clusters comprised both Singapore residents and non-residents. The majority of clusters with MDR-TB also included cases with non-multidrug-resistant MTC isolates (Figure 3).

Patient demographics and distribution of major tuberculosis lineages depending on whether the MTC isolates were clustered by spoligotyping and MIRU-VNTR are shown in Table 2, segregated according to phase of testing. Patients with clustered MTC isolates in both phases were more likely to be of Chinese ethnicity, Singapore resident, and to have an MTC isolate belonging to the Beijing lineage. Patients infected with MTC belonging to the EAI lineage were less likely to be clustered. In the early phase where there was potentially less selective bias, patients with clustered isolates were also more likely to be male and younger in age. MDR-TB isolates were not more likely to belong to a cluster. We have included a brief comparison of cases with MDR-TB vs. those with non-multidrug-resistant MTC as supporting information (Table S2).

**Table 2:** Distribution of patient demographics, susceptibility testing results and major lineages according to combination spoligotyping and MIRU-VNTR clustering status, segregated according to study phase.

**Characteristic**	**Earlier phase (2006-2007)**			**Later phase (2008-2012)**		
	**Clustered isolates** (n = 347)	**Unique isolates** (n = 781)	***p*-value**	**Clustered isolates** (n = 133)	**Unique isolates** (n = 351)	***p*-value**
Median age, years (IQR)	48 (33-60)	50 (32-65)	0.068	45 (30 - 60)	49 (29 - 66)	0.258
Age distribution:			0.032			0.151
0-14 years (%)	0 (0)	4 (0.5)		2 (1.5)	2 (0.6)	
15-64 years (%)	282 (81.3)	584 (74.8)		108 (81.2)	264 (75.2)	
>64 years (%)	65 (18.7)	193 (24.7)		23 (17.3)	85 (24.2)	
Male gender (%)	248 (71.5)	504 (64.5)	0.034	101 (75.9)	243 (69.2)	0.146
Ethnicity:			0.002			0.009
Chinese	226 (65.1)	453 (58.0)		90 (67.7)	207 (59.0)	
Indian	19 (5.5)	68 (8.7)		4 (3.0)	22 (6.3)	
Malay	61 (17.6)	110 (14.1)		23 (17.3)	41 (11.7)	
Others	41 (11.8)	150 (19.2)		16 (12.0)	81 (23.1)	
Singapore resident (%)	269 (77.5)	510 (65.3)	<0.001	103 (77.4)	218 (62.1)	0.001
Multidrug-resistant isolate (%)	11 (3.2)	22 (2.8)	0.707	18 (13.5)	40 (11.4)	0.518
Lineage:			<0.001			<0.001
Beijing (%)	240 (69.2)	253 (32.4)		99 (74.4)	147 (41.9)	
EAI (%)	64 (18.4)	205 (26.2)		22 (16.5)	76 (21.7)	
NEW-1 (%)	7 (2.0)	29 (3.7)		0 (0)	7 (2.0)	
Haarlem (%)	4 (1.1)	16 (2.0)		0 (0)	10 (2.8)	
CAS (%)	0 (0)	17 (2.2)		1 (0.8)	4 (1.1)	

On review of the STEP contact investigations database, there were 386 (80.4%) cases with completed contact investigations, with 120 (90.2%) in the later phase. Only 36 (13 in the later phase) cases belonging to 7 clusters were found to have a prior contact with tuberculosis. Epidemiological links could only be found for cases in 4 (9.5%) of clusters in the later phase. None of the clusters in the earlier or later phases could be completely linked via contact and/or epidemiological investigations. Only a minority of the cases (data not shown) in the later phase for which molecular typing was performed in view of potential epidemiological links (i.e. cases housed in institutional or correctional facilities, or temporally associated cases from workplaces or schools) had identical spoligotyping and MIRU-VNTR results.

On analysis encompassing the entire dataset, 634 isolates could be grouped into 172 identical combined spoligotyping and MIRU-VNTR clusters. There were 12 clusters with at least 10 isolates, and the patients belonging to these clusters had a median age of 46 years (interquartile range: 35-58 years) and were more likely to be male (71.0%), of Chinese ethnicity (65.8%), Singapore resident (80.0%), and have isolates belonging to the Beijing lineage (79.5%). Multidrug-resistant isolates comprise only 0.8% (5 isolates) of this group, and all clusters spanned a minimum of 3 years. The largest cluster comprised 35 patients who presented with tuberculosis between 2006 and 2012. They were largely Singapore residents (80.0%), Chinese (71.4%) and all isolates belonged to the Beijing lineage. Two (5.7%) isolates were multidrug-resistant. 

## Discussion

This large-scale epidemiological study of tuberculosis from Singapore, an Asian city-state with moderate tuberculosis incidence rates, demonstrated tremendous diversity in terms of spoligotyping and MIRU-VNTR profiles. This is unsurprising given that Singapore’s non-resident population currently exceeds 28% of the total population [9], and its expanding medical tourism industry. The major tuberculosis lineages remain the Beijing and EAI lineages, corresponding to earlier work [17]. A combination of MIRU-VNTR typing and spoligotyping was useful for delineating tuberculosis lineages, with 80.1% congruence when compared with spoligotyping alone.

The change in isolate selection strategy for molecular typing between 2006-2007 and 2008-2012 resulted in expected differences in terms of the proportion of isolates that were multidrug-resistant, or belonged to the Beijing lineage. Curiously, despite the selection of proportionately more isolates during the later phase from patients that were deemed to be epidemiologically linked based on contact investigations, the percentage of MTC isolates clustered by molecular typing was similar for both phases. In general, the absolute differences found were not great except in the case of multidrug-resistance, giving an artificially high rate (5.6%) on average. Singapore’s multidrug-resistant tuberculosis rate remains below 1% of all cases of tuberculosis [18]. Nonetheless, the differences were sufficient to suggest significant selection bias, resulting in the decision to keep analyses separate for both study phases.

We had used combined spoligotyping and MIRU-VNTR clustering analysis as a crude proxy for defining local transmission of tuberculosis, with the understanding from recent studies that it is probably not sufficiently discriminatory in accurately defining transmission networks, particularly in geographic settings where the incidence of tuberculosis is relatively high [19,20], or where MTC isolates belonging to the Beijing lineage predominated [21,22]. What was interesting was the finding that patients with clustered isolates in the earlier phase – where there was less selection bias – tended to be younger, Singapore resident and of Chinese ethnicity, although the last is more likely confounded by the Singapore resident status (non-residents with tuberculosis were less likely to be Chinese). Given the long dormancy of tuberculosis infection and the relatively short sampling frame, the cases of late reactivation of tuberculosis in the elderly is less easily clustered, and there may not be sufficient time to detect the scale of infections caused by transmission from non-residents. Nonetheless, the data appears to suggest that cross-transmission between resident and non-resident populations in Singapore had occurred.

In our review of the contact investigation reports at the STEP registry for all cases within clusters, we found no epidemiological links for the majority of patients in every cluster. This failure of current contact investigation strategy to detect epidemiological links is probably due to a combination of three factors. Firstly, molecular typing methods may provide extra capability in highlighting the presence of unknown transmission networks [1,3], particularly if performed in near real-time and on a universal basis as an integral part of contact investigations [1,19]. Secondly, as mentioned above, non-whole-genome sequencing methods are not sufficiently specific and sensitive [6,12,18], and many of the cases in these clusters are not actually related by transmission. Finally, contact investigations have been limited in view of resource constraints, and it is plausible that more extensive investigations would have yielded far more epidemiological linkages. The finding of mixed MDR-TB and non-MDR-TB cases within several recent clusters also supports this, as does the large proportion of MTC isolates belonging to the Beijing lineage, where other investigators have noted that MIRU-VNTR-based clusters could be further and significantly differentiated using IS6110-RFLP [21,22].

There are several other limitations to this work. Except for the initial 2 years, less than 10% of all cultured isolates each year underwent molecular profiling. The isolates from the later phase were also not randomly selected, limiting significantly any detailed conclusions that can be clearly drawn. The short durations of each phase of testing, for which the primary clustering analyses were performed, probably results in an underestimation of the actual number and proportion of case clusters in Singapore [23], and actual chains of transmission are not apparent with the available data. Because of resource constraints, we have not used the results of the molecular typing to re-initiate contact investigations looking for specific links between clustered patients. Nonetheless, the large number of isolates and the wealth of data available do depict the molecular epidemiology of tuberculosis in Singapore to an extent, and allow for broad insights into the local situation. 

Further in depth epidemiological work is required in order to bridge the gaps and validate the findings of our current study. In particular, it is important to identify risk factors for recent transmission and progression to active disease for both the non-resident and resident population, as recent studies have suggested that these factors may be different for different non-resident population groups [24,25]. A case may also be built for the use of whole-genome sequencing rather than spoligotyping and MIRU-VNTR for defining the molecular epidemiology of tuberculosis in Singapore and other countries with moderate-high incidence of tuberculosis, given the better discriminatory power and falling costs of the former.

In summary, our work demonstrates that Singapore is a city-state with a large and heterogeneous distribution of MTC strains, and with possible cross-transmission over the past few years based on our molecular typing results. These findings emphasize the urgent need for enhanced tuberculosis control measures to reduce disease transmission in our country. A universal MTC typing program will be useful in further understanding the transmission dynamics of tuberculosis in Singapore. 

## Supporting Information

Table S1 **Spoligotyping and MIRU-VNTR results for all isolates that were grouped into clusters.**
(XLS)

Table S2 **Distribution of patient demographics and major spoligotypes according to infection by multidrug-resistant MTC.**
(DOC)

## References

[1] Bolotin S, Alexander DC, Guthrie JL, Drews SJ, Jamieson F (2010) The Ontario universal typing of tuberculosis (OUT-TB) surveillance program – what it means to you. Can J Resp J 17: e51-e54. 10.1155/2010/715202 PMC2900146 20617215

[2] Moss AR, Hahn JA, Tulsky JP, Daley CL, Small PM et al. (2000) Tuberculosis in the homeless. A prospective study. Am J Respir Crit Care Med 162: 460-464. doi:10.1164/ajrccm.162.2.9910055. PubMed: 10934071. 10934071

[3] Barnes PF, Cave MD (2003) Molecular epidemiology of tuberculosis. N Engl J Med 349: 1149-1156. doi:10.1056/NEJMra021964. PubMed: 13679530. 13679530

[4] Kamerbeek J, Schouls L, Kolk A, van Agterveld M, van Soolingen D et al. (1997) Simultaneous detection and strain differentiation of *Mycobacterium* *tuberculosis* for diagnosis and epidemiology. J Clin Microbiol 35: 907-914. PubMed: 9157152. 9157152 10.1128/jcm.35.4.907-914.1997 PMC229700

[5] Mazars E, Lesjean S, Banuls AL, Gilbert M, Vincent V et al. (2001) High-resolution minisatellite-based typing as a portable approach to global analysis of *Mycobacterium* *tuberculosis* molecular epidemiology. Proc Natl Acad Sci U S A 98: 1901-1906. doi:10.1073/pnas.98.4.1901. PubMed: 11172048. 11172048 PMC29354

[6] Cardoso Oelemann M, Gomes HM, Willery E, Possuelo L, Batista Lima KV, et al. (2011) The forest behind the tree: phylogenetic exploration of a dominant *Mycobacterium* *tuberculosis* strain lineage from a high tuberculosis burden country. PLOS ONE 6: e18256. doi:10.1371/journal.pone.0018256. PubMed: 21464915. 21464915 PMC3064675

[7] Allix-Béguec C, Fauville-Dufaux M, Supply P (2008) Three-year population-based evaluation of standardized mycobacterial interspersed repetitive-unit-variable-number tandem-repeat typing of *Mycobacterium* *tuberculosis* . J Clin Microbiol 46: 1398-1406. doi:10.1128/JCM.02089-07. PubMed: 18234864. 18234864 PMC2292969

[8] Gardy JL, Johnston JC, Ho Sui SJ, Cook VJ, Shah L et al. (2011) Whole-genome sequencing and social-network analysis of a tuberculosis outbreak. N Engl J Med 364: 730-739. doi:10.1056/NEJMoa1003176. PubMed: 21345102. Available online at: 10.1056/NEJMoa1003176 Available online at: PubMed: 21345102 21345102

[9] Department of Statistics Singapore Population Trends 2012. Available: http://www.singstat.gov.sg/Publications/publications_and_papers/population_and_population_structure/population2012.pdf. Accessed: 15 May 2013

[10] Chee CB, Wang YT (2012) TB control in Singapore: where do we go from here? Singapore Med J 53: 236-238. PubMed: 22511043. 22511043

[11] Ministry of Health Singapore Stop TB in my lifetime. Available: http://www.moh.gov.sg/content/moh_web/pressRoom/pressRoomItemRelease/2012/stop_TB_in_my_lifetime.html. Accessed: 15 May 2013

[12] Chee CB, Gan SH, Chua AP, Wang YT (2012) TB control in Singapore: the high price of diagnostic delay. Singapore Med J 53: 505-507. PubMed: 22941125. 22941125

[13] Sun YJ, Bellamy R, Lee AS, Ng ST, Ravindran S et al. (2004) Use of mycobacterial interspersed repetitive unit-variable-number tandem repeat typing to examine genetic diversity of *Mycobacterium* *tuberculosis* in Singapore. J Clin Microbiol 42: 1986-1993. doi:10.1128/JCM.42.5.1986-1993.2004. PubMed: 15131159. 15131159 PMC404681

[14] Walker TM, Monk P, Grace Smith E, Peto TEA (2013) Contact investigations for outbreaks of *Mycobacterium* *tuberculosis*: advances through whole genome sequencing. Clin Microbiol Infect 19: 796-802. doi:10.1111/1469-0691.12183. PubMed: 23432709. 23432709

[15] Warren R, de Kock M, Engelke E, Myburgh R, van Pittius NC et al. (2006) Safe *Mycobacterium* *tuberculosis* DNA extraction method that does not compromise integrity. J Clin Microbiol 44: 254-256. doi:10.1128/JCM.44.1.254-256.2006. PubMed: 16390984. 16390984 PMC1351970

[16] Allix-Béguec C, Harmsen D, Weniger T, Supply P, Niemann S (2008) Evaluation and user-strategy of MIRU-VNTR*plus*, a multifunctional database for online analysis of genotyping data and phylogenetic identification of *Mycobacterium* *tuberculosis* complex isolates. J Clin Microbial 46: 2692-2699. doi:10.1128/JCM.00540-08. PMC2519508 18550737

[17] Sun YJ, Bellamy R, Lee AS, Ng ST, Ravindran S et al. (2004) Use of mycobacterial interspersed repetitive unit-variable-number tandem repeat typing to examine genetic diversity of *Mycobacterium* *tuberculosis* in Singapore. J Clin Microbiol 42: 1986-1993. doi:10.1128/JCM.42.5.1986-1993.2004. PubMed: 15131159. 15131159 PMC404681

[18] Chee CB, Khin-Mar KW, Cutter J, Wang YT (2012) The imminent threat of multidrug-resistant tuberculosis in Singapore. Singapore Med J 53: 238-240. PubMed: 22511044. 22511044

[19] Hawkey PM, Smith EG, Evans JT, Monk P, Bryan G et al. (2003) Mycobacterial interspersed repetitive unit typing of *Mycobacterium* *tuberculosis* compared to IS6110-based restriction fragment length polymorphism analysis for investigation of apparently clustered cases of tuberculosis. J Clin Microbiol 41: 3514-3520. doi:10.1128/JCM.41.8.3514-3520.2003. PubMed: 12904348. 12904348 PMC179797

[20] Walker TM, Ip CL, Harrell RH, Evans JT, Kapatai G et al. (2013) Whole-genome sequencing to delineate *Mycobacterium* *tuberculosis* outbreaks: a retrospective observational study. Lancet Infect Dis 13: 137-146. doi:10.1016/S1473-3099(12)70277-3. PubMed: 23158499. 23158499 PMC3556524

[21] Hanekom M, van der Spuy GD, Gey van Pittius NC, McEvoy CR, Hoek KG et al. (2008) Discordance between mycobacterial interspersed repetitive-unit-variable-number tandem-repeat typing and IS6110 restriction fragment length polymorphism genotyping for analysis of *Mycobacterium* *tuberculosis* Beijing strains in a setting of high incidence of tuberculosis. J Clin Microbiol 46L: 3338-3345. 10.1128/JCM.00770-08 PMC2566133 18716230

[22] Jiao WW, Mokrousov I, Sun GZ, Guo YJ, Vyazovaya A et al. (2008) Evaluation of new variable-number tandem-repeat systems for typing Mycobacterium tuberculosis with Beijing genotype isolates from Beijing, China. J Clin Microbiol 46: 1045-1049. doi:10.1128/JCM.01869-07. PubMed: 18199785. 18199785 PMC2268385

[23] Martínez-Lirola M, Alonso-Rodriguez N, Sánchez ML, Herranz M, Andrés S et al. (2008) Advanced survey of tuberculosis transmission in a complex socioepidemiologic scenario with a high proportion of cases in immigrants. Clin Infect Dis 47: 8-14. doi:10.1086/588785. PubMed: 18484876. 18484876

[24] Borgdorff MW, van den Hof S, Kalisvaart N, Kremer K, van Soolingen D (2011) Influence of sampling on clustering and associations with risk factors in the molecular epidemiology of tuberculosis. Am J Epidemiol 174: 243-251. doi:10.1093/aje/kwr061. PubMed: 21606233. 21606233

[25] Suwanpimolkul G, Jarlsberg LG, Grinsdale JA, Osmond D, Kawamura LM et al. (2013) Molecular epidemiology of tuberculosis in foreign-born persons in San Francisco. Am J Respir Crit Care Med [Epub ahead of print]. 10.1164/rccm.201212-2239OC PMC3707362 23471470

