# Supplementary material for: Molecular Epidemiology of Mycobacterium tuberculosis Complex in Singapore, 2006-2012
Source: PLoS One. 2013 Dec 18;8(12):e84487. doi: 10.1371/journal.pone.0084487 (PMC3867517; doi:10.1371/journal.pone.0084487)
Supplement: Table S2 — Distribution of patient demographics and major spoligotypes according to infection by multidrug-resistant MTC. (DOC) [file pone.0084487.s002.doc]

Table S2. Distribution of patient demographics and major spoligotypes according to infection by multidrug-resistant MTC.

| **Characteristic** | **Multidrug-resistant MTC** (n = 91) | **Non-multidrug-resistant MTC** (n = 1,521) | ***p-*value** |
| --- | --- | --- | --- |
| Median age, years (IQR) | 33 (27-51) | 49 (32-64) | <0.001 |
| Age distribution: |  |  | <0.001 |
| - 0-14 years (%) | 0 (0) | 7 (0.5) |  |
| - 15-64 years (%) | 86 (94.5) | 1,152 (75.7) |  |
| - >64 years (%) | 5 (5.5) | 361 (23.7) |  |
| Male gender (%) | 38 (41.8) | 471 (31.0) | 0.033 |
| Ethnicity: |  |  | <0.001 |
| - Chinese | 42 (46.2) | 935 (61.4) |  |
| - Indian | 5 (5.5) | 108 (7.1) |  |
| - Malay | 5 (5.5) | 230 (15.1) |  |
| - Others | 39 (42.9) | 247 (16.2) |  |
| Singapore resident (%) | 35 (38.5) | 1,065 (70.0) | <0.001 |
| Lineage: |  |  | 0.005 |
| - Beijing lineage (%) | 60 (65.9) | 679 (44.6) |  |
| - EAI lineage (%) | 12 (13.2) | 355 (23.3) |  |
| - NEW-1 (%) | 3 (3.3) | 40 (2.6) |  |
| - Haarlem (%) | 2 (2.2) | 38 (2.5) |  |
| - CAS lineage (%) | 1 (1.1) | 21 (1.4) |  |
